# Partial Recovery of Anosmia Since Childhood With Acquired Parosmia: Mechanistic Insights and Therapeutic Implications—A Case Report

**DOI:** 10.1155/crot/7811132

**Published:** 2026-03-10

**Authors:** Eishaan Kamta Bhargava, Shubin Li, Yling Mai, Susanne Weise, Antje Hähner, Thomas Hummel

**Affiliations:** ^1^ Smell and Taste Clinic, Technische Universität Dresden, Dresden, Germany, tu-dresden.de; ^2^ Department of ENT, Sheffield Children’s Hospital, Sheffield, UK

**Keywords:** corticosteroids, neurogenesis, olfactory bulb, olfactory dysfunction, olfactory epithelium, olfactory pathways, smell disorders

## Abstract

**Background:**

Isolated congenital anosmia (ICA) traditionally represents an irreversible sensory disorder affecting 1 in 5,000–10,000 individuals. This case demonstrates rare partial olfactory recovery in childhood‐onset anosmia following corticosteroid therapy, providing novel insights into olfactory neuroplasticity and therapeutic potential.

**Case Presentation:**

A 32‐year‐old woman with lifelong anosmia, diagnosed with ICA at age 20 using Sniffin’ Sticks testing (2013), experienced first olfactory perceptions in 2014 following one week of oral corticosteroids and nine months of topical mometasone. MRI (2021) revealed barely visible olfactory bulbs, narrow olfactory clefts, minor ethmoid sinus inflammation, but normal‐depth olfactory sulci and typical skull base morphology. Recent psychophysical testing (2025) demonstrated orthonasal hyposmia (TDI score 24) and retronasal hyposmia (11/20). Electrophysiological assessment detected preserved olfactory event–related potentials to hydrogen sulfide and phenyl ethyl alcohol, confirming functional neural circuitry. Recovery was complicated by parosmia, with foods like garlic, eggs and meat perceived as intensely unpleasant and faecal‐smelling, leading to dietary modifications and preference for low‐odour foods. Patient reported childhood social difficulties related to anosmia, with ongoing evolution of olfactory function over the past decade. Current treatment includes tapered systemic and topical corticosteroids, intranasal vitamin A drops (10,000 IU/day) and olfactory training, with recent improvements including tolerance of previously aversive foods.

**Conclusions:**

This case challenges the irreversibility paradigm of childhood‐onset anosmia and demonstrates that olfactory recovery is possible even after decades, potentially mediated by anti‐inflammatory effects and neurogenic plasticity. The emergence of parosmia during recovery reflects maladaptive neuroplasticity during olfactory regeneration. Combined corticosteroid therapy, vitamin A supplementation and olfactory training may offer therapeutic hope for similar patients, warranting further investigation of inflammatory modulation and neuroregenerative approaches in congenital olfactory disorders.

**Key Points**
• Partial olfactory function recovery is possible in anosmia since childhood.
• Parosmia may emerge during recovery, reflecting maladaptive olfactory neuroplasticity.
• Anti‐inflammatory and neuroregenerative therapies offer hope for functional rehabilitation.
• Preserved olfactory circuitry can exist despite hypoplastic olfactory bulbs on MRI.

## 1. Introduction

Anosmia since birth is a rare, traditionally irreversible disorder, affecting an estimated 1 in 5000 to 1 in 10000 individuals [1]. We present a case of anosmia since childhood with partial olfactory recovery and parosmia following corticosteroid therapy. This case offers insights into olfactory neurogenesis, the role of inflammatory processes in perpetuating olfactory dysfunction and the potential for neuroregeneration even after decades of sensory loss.

## 2. Methods

This clinical experience is reported in line with CARE guidelines on clinical case reporting [2, 3]. Informed consent was obtained from the patient, and the report was deemed exempt from ethical committee review due to the anonymised nature of the case report.

## 3. Case Presentation

**FIGURE 1:**
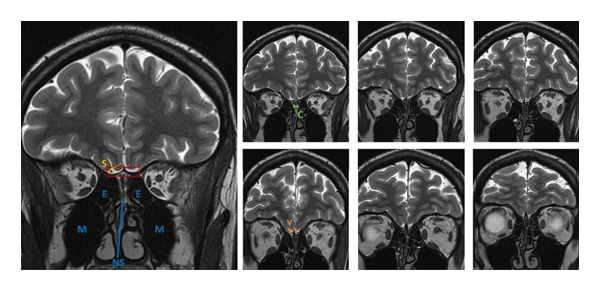
MRI scan with hypoplastic olfactory bulbs, normal depth olfactory clefts and minor inflammatory changes in the ethmoid sinuses. Note: S: olfactory sulcus; E: ethmoid sinuses; M: maxillary sinuses; NS: nasal septum; C: olfactory clefts; V: vessels; red rectangle: typical location of the olfactory bulbs, although they are not visible in this case.

A 32‐year‐old woman reported lifelong anosmia, diagnosed as isolated congenital anosmia (ICA) at age 20 years following psychophysical testing for smell using Sniffin’ Sticks in 2013 (detailed threshold, discrimination and identification subscores were not available). In 2014, at age 21, she was prescribed a course of systemic corticosteroids followed by topical mometasone therapy. While the specific clinical indication, dosing and delivery technique for this treatment were not documented in available records, the patient reported consistent use and noted that approximately 3‐4 weeks after initiating treatment, she experienced her first conscious olfactory perceptions. An MRI in 2021 (Figure 1) revealed barely visible olfactory bulbs (OBs) consistent with ICA [4]. There were minor inflammatory changes in the ethmoid sinuses and narrow olfactory clefts, suggesting a mixed aetiology of developmental olfactory system anomaly with superimposed inflammatory changes. The skull base morphology on MRI was typical of normosmic people with fully developed OBs [5]. Olfactory sulci were of normal depth. Recent psychophysical testing (2025) showed orthonasal (Sniffin’ Sticks TDI score 24; *T* = 1, *D* = 10, and I = 13), and retronasal hyposmia (11/20). Gustatory testing and nasal endoscopy were normal.

Electrophysiological assessment was carried out using the electrobulbogram (EBG) methodology [6], with olfactory event–related potentials (OERPs) recorded via EEG electrodes positioned at the nasal bridge. Testing detected OERPs to hydrogen sulfide and phenyl ethyl alcohol (Figure 2), with characteristic high‐frequency gamma‐band responses (> 70 Hz) within 100–200 milliseconds post‐stimulus onset, indicating preserved olfactory neural circuitry. The patient was advised to try tapered systemic plus topical corticosteroids, vitamin A‐containing nasal drops (10000 IU/d) and olfactory training.

FIGURE 2Time‐frequency analysis graphic depicting detectable OERPs recorded from the electrobulbogram (EBG) to phenyl ethyl alcohol (a) and hydrogen sulfide (b). Note: The plots display frequency (10–100 Hz) over a 1 s time window following stimulus onset. Red ellipses highlight time‐frequency clusters of interest. Both stimuli elicited distinct high‐frequency responses in the gamma band (> 70 Hz), with additional low‐frequency activity.

**Figure:**
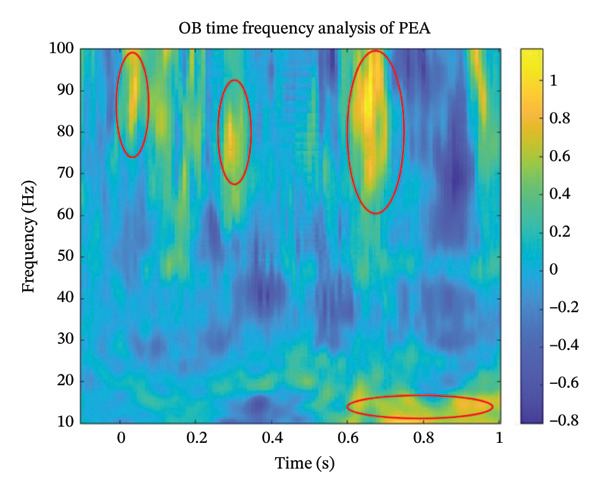
(a)

**Figure:**
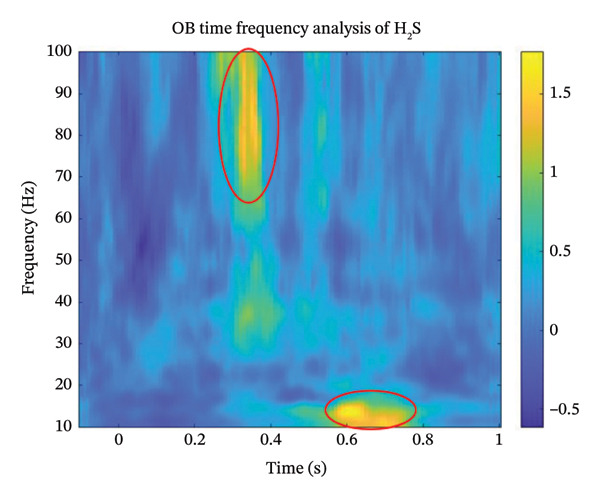
(b)

### 3.1. Patient‐Reported Experiences

The patient described childhood social exclusion due to anosmia, with difficulty relating to social cues involving smell, and reliance on food texture for dietary choices. After initial systemic and topical corticosteroid therapy, she experienced a ‘blurry,’ fragmented emergence of smell complicated by parosmia. Foods like garlic, eggs and meat became intensely unpleasant, described as faecal in odour, shaping her aversion to complex or heavily spiced cuisines and preference for foods with lower odour intensity, like Japanese rice–based dishes.

Despite a decade of gradual recovery, her olfactory function is still evolving. She is only now beginning to appreciate the nuances and associations of different scents and flavours. Since starting the intranasal treatment with vitamin A‐containing nasal drops and olfactory training outlined above, she has come to appreciate and love the scent and flavour of tarragon and is now able to tolerate filet mignon. Negative associations with newly discovered strong smells such as sweat and sewage continue to predominate her olfactory recovery journey. She describes her experience as predominantly negative, mostly due to the sudden change in her sensory experience, but remains hopeful for improvement.

## 4. Discussion

The coexistence of ICA hallmarks (hypoplastic OBs and lifelong anosmia) with inflammatory imaging features (ethmoid sinus mucosal changes and narrow olfactory clefts) and a response to corticosteroids suggests a possible mixed pathophysiology combining developmental and acquired components, with chronic low‐grade inflammatory component potentially suppressing function of residual sensory elements. Such inflammatory changes may also lead to a hypoplastic OB, so her anosmia into early adulthood may have been inflammatory. The presence of normal‐depth olfactory sulci on MRI [1] and regular morphology of the skull base [5], in conjunction with the objective evidence of functional olfactory circuitry on electrophysiological assessment, may indicate normal olfactory system development at birth, despite poorly developed OBs. This aligns with recent population‐based data demonstrating that preserved olfactory function can exist without anatomically defined OBs on imaging, with Weiss et al. identifying approximately 0.6% of individuals in a large neuroimaging database who demonstrated normal olfactory psychophysical performance and typical piriform cortex fMRI activation despite the complete absence of structurally defined OBs [7].

### 4.1. Mechanistic Insights

1. Steroid‐induced neurogenic priming: Recent evidence supports adult human olfactory neurogenesis [8], with neurogenic niches in olfactory epithelium. Corticosteroid therapy may suppress subclinical inflammation, enabling dormant progenitors to differentiate.
2. Maladaptive plasticity and parosmia: Parosmia is common in early recovery from postviral or posttraumatic anosmia, often triggered by foods containing thiols, pyrazines and disulfides [9]. Incomplete characterization of odorants with predominant detection of these compounds at concentrations below normal detection thresholds [9] may underlie some distortions during olfactory neuron regeneration. This, alongside a sudden shift in sensory experience without gradual learnt social and emotional responses to odours [10], may explain the patient’s aversion to certain foods. The specific characterization of parosmic sensations as ‘faecal’ despite lifelong anosmia represents a fascinating phenomenon. This may be due to a combination of semantic characterisation of novel sensations following acquisition of conceptual knowledge of odour descriptors through language and social observation without direct olfactory experience, innate aversive responses to thiols and sulphur compounds (a major faecal odour component) and affective mapping of descriptors based on emotional valence (intense displeasure) rather than true olfactory similarity.
3. Anti‐inflammatory effects of corticosteroids: Even after decades of anosmia, systemic corticosteroids followed by topical application may restore some olfactory function.

### 4.2. Therapeutic Implications

1. Inflammatory modulation: Systemic and topical corticosteroids in anosmic patients with detectable OBs and normal olfactory sulci may promote neurogeneration through reduction of mucosal oedema in the olfactory cleft, lowering inflammatory cytokines that may suppress neurogenesis and creating a microenvironment permissive for olfactory neuronal maturation.
2. Enhanced recovery protocols: Retinoic acid has been implicated in olfactory neuron differentiation; intranasal Vitamin A combined with olfactory training may improve olfactory function [11].
3. Dietary modulation of parosmia: Understanding molecular triggers [9] may guide dietary interventions to alleviate negative experiences and improve recovery.

To our knowledge, only one other case has reported olfactory recovery in presumed ICA [10]. The key similarities and differences to our case are outlined in Table 1. The superior functional trajectory in our case may reflect therapeutic benefit of anti‐inflammatory intervention, active rehabilitation and extended neuroplastic adaptation.

**TABLE 1:** Comparison of olfactory recovery cases in lifelong anosmia.

Feature	Current case	Pellegrino et al. [10]
*Demographics and presentation*		
Age at diagnosis (years)	20	13
Age at recovery onset (years)	21	24
Sex	Female	Female
Family history	Negative	Negative

*Baseline olfactory function*		
Initial diagnosis	Functional anosmia	Functional anosmia
Baseline TDI score	Not documented	Unable to detect 12 test odours
Retronasal function at baseline	Not assessed	Not assessed

*Neuroimaging findings*		
Olfactory bulbs	Barely visible bilaterally	Absent bilaterally
Olfactory sulcus depth	Normal bilaterally	Flattened on left; clear but flattened on right
Skull base morphology	Normal	Not mentioned
Additional findings	Minor ethmoid sinus inflammation; narrow olfactory clefts	None reported

*Recovery characteristics*		
Trigger/intervention	Systemic + topical corticosteroids	Spontaneous (no specific intervention)
Onset timing	3‐4 weeks after steroid initiation	Gradual over months starting age 24
Recovery pattern	Faint initial perceptions, gradual intensification with parosmia	Fragmented emergence with phantosmia

*Postrecovery olfactory function*		
Evaluation timepoint	11 years postrecovery (2025)	1–3 years postrecovery
Orthonasal TDI score	24 (hyposmia)	12 (functional anosmia)
Threshold (T)	1	1
Discrimination (D)	10	5
‐ Identification (I)	13	6
Retronasal function (score/total)	11/20 (hyposmia)	15/20, later 12/19
Subjective odour detection	Multiple odours; evolving repertoire	16/32 test odours (50%)

*Electrophysiological assessment*		
OERP/EBG to H_2_S	Present	Present
OERP/EBG to PEA	Present	Present
Interpretation	Preserved functional circuitry	Intact olfactory system despite absent bulbs

*Qualitative distortions*		
Type	Parosmia	Phantosmia (lingering odours)
Descriptors	‘Faecal;’ intensely unpleasant	‘Cannot switch off;’ stressful
Trigger foods	Garlic, eggs, meat	Trigeminal‐component odours
Impact	Dietary modifications; food aversions	Disturbing; ‘annoyance’

*Patient experience*		
Hedonic valence	Predominantly negative initially; improving	Disturbing; negative
Social/emotional impact	Childhood social difficulties; ongoing challenges improving	Stress from persistent sensations
Recovery trajectory	Gradual improvement over 11 years with training	Evolution ongoing at time of report

*Current treatment*		
Active interventions	Tapered systemic corticosteroids followed by topical corticosteroids; intranasal vitamin A (10,000 IU/day); olfactory training	None reported
Treatment response	Improved tolerance; some foods now enjoyable (e.g., tarragon and filet mignon)	Not applicable

*Proposed mechanisms*		
Primary hypothesis	Anti‐inflammatory + neurogenic plasticity	Hormonal influences on delayed neurogenesis
Supporting evidence	Response to steroids; inflammatory imaging findings; normal olfactory sulci	Spontaneous onset; age‐related factors

## 5. Conclusions

This case challenges the traditional view that anosmia since childhood is an irreversible condition and demonstrates that it may exist on a spectrum influenced by inflammatory and neurogenic plasticity. While corticosteroids and olfactory training are the mainstay of treatment, dietary modulation to cope with unpleasant odorous sensations and emerging therapies such as intranasal vitamin A may offer hope for improved outcomes.

Potential areas of future research with a personalised medicine focus may be olfactory cleft cytokine profiling to predict therapeutic response and investigating the genetic basis of parosmia susceptibility.

## Author Contributions

Eishaan Kamta Bhargava: substantial contributions to conception, design, drafting and critical revision and final approval of submitted manuscript; Shubin Li, Yling Mai, and Susanne Weise: substantial contributions to conception, data acquisition, critical revision and final approval of submitted manuscript; Antje Hähner and Thomas Hummel: substantial contributions to conception, critical revision and final approval of submitted manuscript.

## Funding

No sources of funding to disclose.

## Disclosure

All authors agree to be accountable for all aspects of the work.

## Ethics Statement

Informed consent was obtained from the patient (available on request), and the report was deemed exempt from ethical committee review due to the anonymised nature of the case report.

## Conflicts of Interest

Since 2023, Thomas Hummel collaborates with the following companies: Sony, Tokyo, Japan; Smell and Taste Lab, Geneva, Switzerland; Takasago, Paris, France; Cyrano, Delray Beach, FL, USA; Cynexo, Trieste, Italy; Sentosphere, Paris, France; NOAR, Sao Paulo, Brazil; Burghart, Holm, Germany; Rhino Therapeutics, Boston, MA, USA. The remaining authors declare no conflicts of interest.

## Data Availability

The data that support the findings of this study are available on request from the corresponding author. The data are not publicly available due to privacy or ethical restrictions.
